# Mechanical Behaviour and Induced Microstructural Development upon Simultaneous and Balanced Biaxial Stretching of Poly(ethylene furandicarboxylate), PEF

**DOI:** 10.3390/polym15030661

**Published:** 2023-01-28

**Authors:** Emilie Forestier, Christelle Combeaud, Nathanaël Guigo, Nicolas Sbirrazzuoli

**Affiliations:** 1 Centre for Material Forming (CEMEF), Mines Paris, PSL University, UMR CNRS 7635, 06904 Sophia Antipolis, France; 2 Institut de Chimie de Nice (ICN), Université Cote d’Azur, CNRS, UMR7272, CEDEX 2, 06108 Nice, France

**Keywords:** Polyethylene 2,5-furandicarboxylate, biaxial stretching, microstructural development, strain-induced crystallization (SIC)

## Abstract

The biaxial behavior of PEF has been analyzed for equilibrated and simultaneous biaxial stretching. The ability of PEF to develop an organized microstructure through strain induced crystallization (*SIC*) has been described. Upon biaxial stretching, *SIC* can be difficult to perform because the stretching is performed in two perpendicular directions. However, thanks to the time/temperature superposition principle and an accurate heating protocol, relevant stretching settings have been chosen to stretch the material in its rubbery-like state and to reach high levels of deformation. By the protocol applied, the mechanical behavior is easily transposable to the industry. This work has shown that PEF can, as in uniaxial stretching, develop well-organized crystals and a defined microstructure upon biaxial stretching. This microstructure allows the obtention of improved mechanical properties and thermal stability of the biaxially stretched samples. The crystals induced upon biaxial stretching are similar to the one that has been developed and observed after uniaxial stretching and upon static crystallization. Moreover, the furan cycles seem to appear in a state similar to the one of a sample crystallized upon quiescent condition. The rigidity is increased, and the *α*-relaxation temperature is increased by 15 °C.

## 1. Introduction

Poly(ethylene 2,5-furandicarboxylate), PEF, is a biobased polymer which has gained more and more interest over the last decade [1,2,3,4,5,6,7,8,9]. This polyester remains one of the best solutions to offer a biobased alternative to PET in food packaging, or other applications that require a fibre texture [10,11,12]. PET mainly comes from petroleum resources and exhibits good mechanical, thermal and barrier properties. Accordingly, the processes of bottle blowing or thermoforming have evolved thanks to PET features [13,14]. PEF and PET are structurally similar excluding the presence of a furan cycle in PEF, composed of four carbons and 1 oxygen atom with two non-binding electrons, which leads to a hindered chain mobility for PEF [15,16].

For implementing a new material in the market, it is important to understand the way of processing it, and to determine its barriers properties, as well as its mechanical and thermal behaviors upon quiescent conditions. It has been mainly reported that PEF is more rigid than PET, exhibits a slower crystallization rate and presents better barrier properties [15,16,17,18,19,20,21,22,23,24,25,26,27,28,29,30]. From this understanding, and to fit with industrial processes, such as bottle blowing, the mechanical behavior and the associated microstructural development upon uniaxial and biaxial stretching have been explored recently [31,32,33,34,35,36,37,38,39,40]. The correct monitoring of the stretching test is crucial to ensure the development of a stable and crystalline microstructure upon stretching and industrial process. This phenomenon is known as strain induced crystallization, or “*SIC*”. When PEF and PET were compared upon uniaxial stretching, the investigation revealed that PEF, like PET, was able to develop a well-organized microstructure, only if relevant stretching conditions were selected [35,38]. The PET chain is composed of ethylene glycols in *gauche* or *trans* conformations. When PET is uniaxially stretched, the chains are extended and the ethylene glycols in *trans* conformations are getting more numerous, while the number of ethylene glycols in *gauche* conformation is decreasing [41]. On the other side, PEF chains are composed of ethylene glycols in *gauche* and *trans* conformations, but also in furan cycles in *syn* and *anti* conformations. Upon PEF stretching, as in PET, the number of ethylene glycols in *trans* conformation is increasing, but additionally there is also an increase of furan cycles in *syn* conformation [30]. After the reaching of a certain level of deformation, crystals appear [42,43]. An annealing step is helpful to improve the microstructural development for PET. Moreover, upon uniaxial stretching PEF forms crystals before the occurrence of the strain hardening, while PET develops firstly an intermediary phase named “mesophase” [37]. The presence of this mesophase leads to more diversity in the microstructure developed, in comparison to PEF which exhibits a really similar structure without a strong impact of the stretching settings [35].

Then, this paper explores the PEF mechanical behavior upon biaxial stretching with the objective of developing a microstructure that will lead to an improvement of the properties of the amorphous sample. PET biaxial stretching has been widely explored over the last years [14,42,44,45,46,47,48,49,50,51,52,53,54,55,56,57,58,59]. PET induces an organized microstructure upon biaxial solicitation that is equivalent to what can be observed after uniaxial stretching [60]. Upon biaxial elongation, the phenyl rings can organize themselves in the plane of the film, building up a packed and arranged microstructure in the thickness of the specimen [52]. Because of this arrangement, barrier properties are then improved.

According to the authors knowledge, only two papers deal with PEF biaxial stretching [32,40], and report that the stretching of PEF in biaxial conditions is possible even if its strain hardening is difficult to occur. Upon biaxial stretching, PEF strain hardening starts at higher strains in comparison to what is observed for PET [40]. This feature has also been reported for PEF towards uniaxial stretching tests [37,38]. Moreover, the impact of the molecular weight of PEF upon biaxial stretching has been studied by Stoclet et al. They concluded that PEF strain induced crystallization upon biaxial stretching is easier when the molecular weight increases, and that a sufficient orientation level must exist. A high molecular weight favors strain induced crystallization. However, a low crystallinity ratio is reported upon biaxial stretching in this work. These authors suggest that the extension of the chains leads to the strain hardening occurrence [32].

In the work described in this paper, an approach that relies on simultaneous and balanced tests has been proposed: the elongation is applied in the two orthogonal directions until the same final stretching ratios. An original protocol that is based on the construction of master curve to select relevant stretching settings has been applied for PEF biaxial stretching. This efficient protocol, validated for PEF uniaxial stretching, allows the selection of stretching settings that consider the mobility of the chains, in relation with the physical state of the material [38]. Then, the induction of a stable and well-defined microstructure, which is of prime interest for the further commercialization of PEF, is expected. Additionally, to perform isothermal biaxial stretching tests, the heating protocol was carefully settled. The control of the temperature during the test is of prime importance when *SIC* is researched. Two different post-stretching treatments have been performed on the stretched samples: a quenching step and an annealing step during 3 min at the temperature of elongation. The post stretching properties, both issuing from the quenching or annealing steps, were then investigated. The microstructure developed after biaxial stretching tests is compared to what is reported after an efficient uniaxial stretching.

## 2. Materials and Methods

### 2.1. Materials

The synthesis of poly(ethylene 2,5-furandicarboxylate) was performed from direct esterification. The molecular weight was increased, by Avantium Renewable Polymers, by a two steps process of melt-state followed by solid-state polycondensation of monoethylene glycol and 2,5-furandicarboxylic acid (FDCA). The sheets were extruded, and the thickness was 700 μm. Samples were taken off in the extrusion direction. Samples were stored under vacuum, in an aluminum coated bag, in the freezer (−18 °C) to avoid water absorption and physical aging. Consequently, the polymer was tested dry as processed, without any pre-conditioning.

### 2.2. X-ray Scattering

Wide-angle X-ray scattering (WAXS) experiments were conducted in order to describe the “organized” or crystalline phases. Debye–Scherrer patterns (2D), with the flat-film camera technique under vacuum at ambient temperature, have been acquired. The sample-screen distance was set to 75 mm. The exposure time was of 45 min. 1D scans, *I(2θ)*, are also performed at room temperature, in transmission and reflexion mode (from 10° to 50°), using a diffractometer Philips X’Pert PRO supplied by Panalytical. For the transmission, the scan intensities were normalized by the sample thickness. In both cases, the CuKα radiation (*λ_CuKα_* = 1.54 Å) was used.

### 2.3. DMTA Measurements

DMTA experiments were conducted in tension mode, using a Mettler-Toledo^®^ DMA 1 (Zurich, Switzerland). The sample dimensions were 5 mm, 4 mm, and 0.7 mm, respectively, for the length, the width, and the thickness for the non-stretched amorphous sample. In the case of the biaxially stretched samples, the dimensions were approximately 5 × 10 × 0.3 mm^3^. The preload applied was of 1 N. Temperature scans were realized at a frequency of 1 Hz from −150 °C and 200 °C, at a heating rate of 1 °C/min, with a displacement amplitude of 5 μm (i.e., strain of 0.1%), in Auto-Tension mode.

Upon heating, thermal dilatation or shrinkage can occur, resulting from a change in the sample length. These changes can be estimated by DMTA by measuring the evolution of the static displacement (Δ*L*) through the calculation of the thermal deformation (Equation (1)).

(1) $$Deformation\left(\%\right)=\frac{\Delta L}{L_{0}}\times 100$$

with *L*_0_ the initial sample length, and Δ*L* the length variation.

### 2.4. DSC Measurements

By using a Mettler Toledo DSC 1 equipped with the STAR^®^ software, DSC measurements were carried out in crucibles made of aluminum (40 µL). Biaxially stretched samples are extremely thin, so the sample weight was approximately 1 mg. The enthalpy uncertainness is then evaluated at around 5%.

The stretched samples were heated from 70 °C to 250 °C at a heating rate of 10 °C/min. Crystallinity ratios have been calculated according to Equation (2):

(2) $$\chi_{SIC}=\frac{{\Delta H}_{m}-{\Delta H}_{c}}{{\Delta H}_{m}^{0}}$$

where *χ_SIC_* is the crystallinity ratio induced by the stretching, Δ*H_m_* the melting enthalpy, Δ*H*_c_ the cold crystallization enthalpy, and Δ*H_m_*^0^ the equilibrium melting enthalpy, taken at 140 J·g^−1^ for PEF and PET as used elsewhere [25,27,38].

### 2.5. FT-IR Measurement

A Bruker TENSOR 27^®^ spectrophotometer in ATR (absorbance mode) with a diamond crystal was used in a range varying from 4000 cm^−1^ to 600 cm^−1^, 64 scans were cumulated, and the resolution is of 4 cm^−1^. A normalization of the spectra by the maximum of the band localized at 1216 cm^−1^ was applied. This band is related to the ester groups of the amorphous sample.

### 2.6. Stretching Protocol

By firstly using an analysis of PEF mechanical behavior in the low deformation domain by DMTA, accurate stretching parameters (strain rate/temperature) can be found. The efficiency of this protocol has already been presented in a previous paper [38]. The maximum of the *Tan δ* peak gives the *α*-relaxation temperature which is reported at 92 °C for a measurement performed with 1 Hz frequency, while the cold crystallization starts around 155 °C.

**Figure 1:**
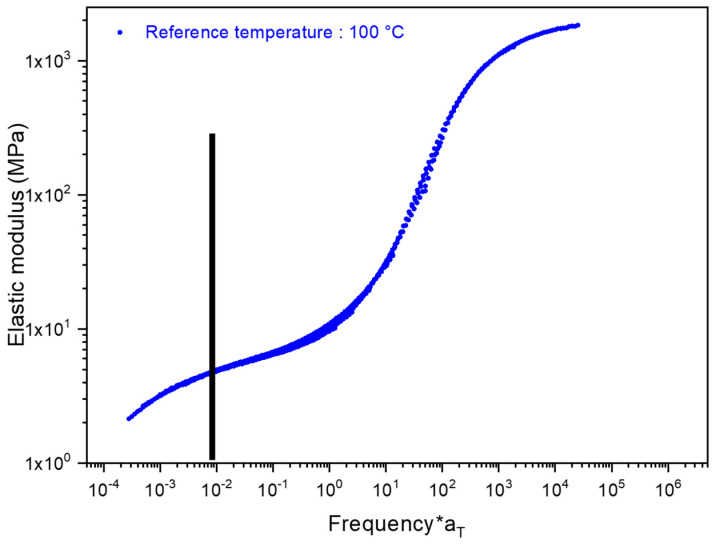
PEF master curve obtained for a reference temperature of 100 °C. The stretching settings have been selected from a equivalent strain rate of 10^−2^ s^−1^ represented by the black line.

Then, according to the stretching protocol used, the PEF master curve is built for a reference temperature chosen close to the *α*-relaxation temperature, here 100 °C (Figure 1). More details relative to the building of the master curve can be found in the following papers [38,39].

The mechanical response of the material upon stretching is governed by the choice of the localization on the master curve. This position determines the existing gap from the *α*-relaxation transition, and then the physical state of the samples and the mobility of the chains. This gap is represented by the equivalent strain rate, that lead to the determination of a couple strain rate/temperature [38]. In this work, the chosen localization is in the middle of the rubbery plateau where chains have a high mobility, and can be stretched up to high deformations, if the stretching settings are accurately chosen. Figure S1 in Supporting Information depicts the geometry and features of the biaxial samples.

The biaxial stretching tests were performed on a homemade device, with controlled temperature and strain rate. This device allows to reproduce precisely the thermomechanical solicitations that exist in industrial conditions, such as bottles blowing. A deeper description of the device can be found in [61]. The device is designed with four independent, motor-driven arms, each coupled to a displacement sensor and a 500 N force transducer. The tests were conducted to keep the strain rate, *ἐ*_0_, constant in the process zone of the samples. An exponential velocity was applied to control the displacement of each arm.

To measure the evolution of the specimen surface temperature upon stretching, the oven is composed of a window made with zinc selenide (ZnSe), which is partially transparent to infrared radiation. The second window is made with borosilicate glass, with the aim of measuring the second side of the sample that is painted with a speckle. Local measurements of strain fields using DIC (2D Digital Image Correlation) were then possible. For each measurement, an infra-red pyrometer and a CCD camera were synchronized to the other analogic signals (force, displacement…). Then, true stress/strain curves can be obtained. It was checked that a speckle of a thickness of around 40 µm did not impact the force measurement. The local Hencky’s strain was measured on the sample diagonal (*ε_diagonal_*) with DIC2D.

The deformation is homogeneous on the diagonal of the sample because the loading is balanced. By this way, the Hencky’s strain is averaged on the diagonal. Figure S2a shows the evolution of the strain on the two diagonals with time, while Figure S2b represents the evolution of the typical Hencky’s strains during the test. The values are also gathered it Table 1. It was checked that the material was mechanically isotropic. Then, both force measurements were equivalent in both directions. Equation (3) displays the calculation of the true stress.

(3) $$\sigma \left(t\right)=\frac{\sqrt{2}F(t)}{e_{0}*L_{diagonal}*e^{-\varepsilon_{diagonal}}}$$

with *σ(t)* the true stress, *F*(t) the force measured in one direction, *e*_0_ the initial thickness, *L_diagonal_* the diagonal length, and *ε_diagonal_* the Hencky’s strain on the diagonal.

To obtain biaxial specimens that can be microstructurally analyzed, it has been decided to not continue the tests up to the rupture of samples. The values of the global stretching ratio (*λ_biaxial_*) and local strain measurements are calculated from the values of the Hencky’s strains, as described in Equations (4) and (5), and gathered in Table 1.

To perform microstructural analysis in the process zone after the biaxial elongation, the speckle was mechanically removed. As a reminder, the local stress and strain were measured in this area.

(4) $$\lambda_{biaxial}=\lambda_{diagonal1}*\lambda_{diagonal2}$$

(5) $$\lambda_{diagonal1}=\exp\left(\varepsilon_{diagonal1}\right)$$

where *λ_diagonal_*_1_ and *λ_diagonal_*_2_ represent the draw ratio on each diagonal, and *ε_diagonal_*_1_ the Hencky’s strain on one diagonal. The same calculation is made on the other diagonal.

According to the high biaxial draw ratio obtained, it can be observed that PEF is able to be highly stretched upon biaxial solicitation before rupture. The Hencky’s strains appear relatively similar in all the directions, supporting the good monitoring of the mechanical tests performed.

### 2.7. Heating Protocol and Post-Stretching Treatment

Because of the shape of the biaxial samples and the clamps (Figure S1), an homogeneous heating by convection is not easy to impose. Indeed, it can lead to a localization of the deformation close to the clamping zones that are designed with smaller cross-sections. To localize the deformation in the central zone, it is then necessary to weaken it. According to previous work [43], a thermal weakening has been applied. By this way, a heating pinch was used at a temperature *T_pinch_,* for 3 min, to ensure a more local heating of the specimen by conduction. After this step, the pinch is removed and a hot oven, at a temperature *T_oven_* (*T_oven_* < *T_pinch_*), is positioned above the sample. Thanks to this heating protocol, the center of the sample is heated up until a temperature higher than the glass temperature one, while the clamping zones remain glassy. The process zone is then submitted to local and uniform deformation while avoiding the stretching of the specimen arms. During all the tests, the surface temperature was recorded in the center of the sample. As a result, the equivalent strain rate at a reference temperature, which is the controlling parameter of this protocol, can be known in real time.

To analyze the microstructural changes due to the mechanical tests, two post-stretching treatments were conducted: one sample is quenched with cold-air (the quenching rate is approximatively of −1000 °C/min), while the second one is annealed during 3 min at 140 °C, which corresponds to the oven temperature.

### 2.8. Evolution of the Strain Rate, the Temperature and the Equivalent Strain Rate

The following paragraphs are describing the mechanical analysis, to clarify the plotting curves only the “quenched test” is presented. The “annealed test” is mechanically equivalent to the previous one, proving the good reproducibility of the tests performed.

**Figure 2:**
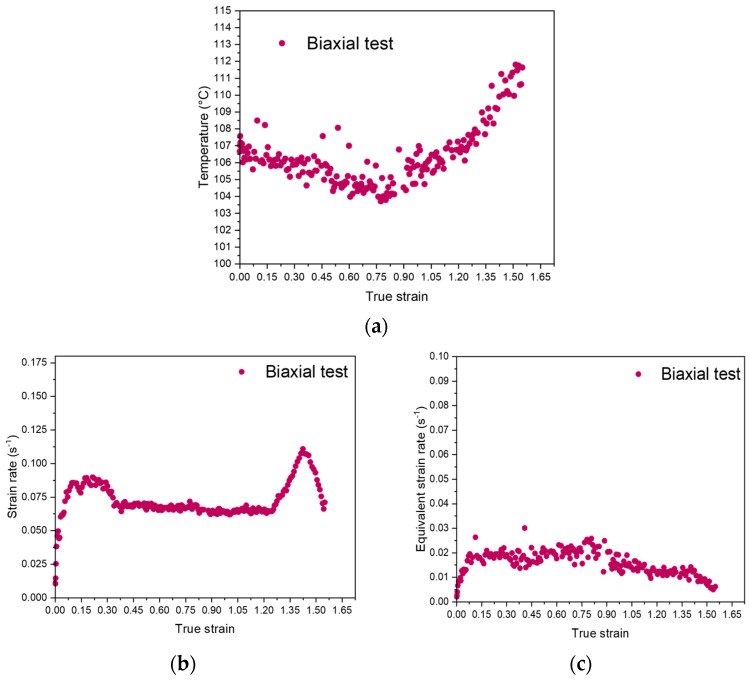
Evolution of (**a**) the temperature, (**b**) the strain rate and (**c**) the equivalent strain rate at a reference temperature of 100 °C, in equilibrated biaxial conditions.

The evolution of the temperature, the strain rate, and the equivalent strain rate during the biaxial tests is represented in respectively Figure 2a–c.

In the previous figures, it can be observed that the temperature and the strain rate can remain relatively constant during biaxial tensile tests, up to the onset of strain hardening. The values for the strain rate and the temperature are respectively of 0.065 s^−1^ and of 105 °C. The equivalent strain rate appears relatively constant (it remains in the same decade), and is of 0.02 s^−1^ for a reference temperature of 100 °C. The measured value is the one expected, as presented in Figure 1 by the black line. By this way, the stretching parameters selected are fitting with a stretching performed in the middle of the rubbery plateau. An increase of the temperature is observed for a true strain of 0.9. As proposed in a previous work focused on uniaxial stretching, this increase can be due to crystallization, which is an exothermic phenomenon, or to self-heating, or to a combination of both phenomena [35]. The previous observation explains the evolution of the equivalent strain rate (Figure 2c) defined at the reference temperature of 100 °C. With the strain-hardening occurrence, the material becomes harder and harder to deform, which may explain the slight decrease of the equivalent strain rate during the last stages of the stretching.

Finally, the temperature of the biaxial tests presented in this work is higher in comparison to what has been tested in previous works made by other groups [32,40]: it should have an influence on the microstructural development. The parameters chosen in the presented work allow the polymer to optimize the chain mobility, while permitting experiments that can be transposed to the industry because of the stretching protocol defined.

## 3. Results and Discussion

### 3.1. Analysis of the Mechanical Behavior

**Figure 3:**
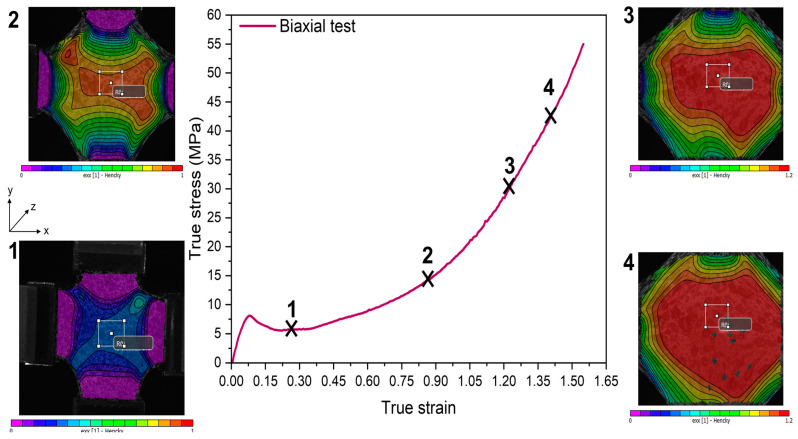
True stress strain curve of PEF upon equilibrated and balanced biaxial stretching, at a temperature of 105 °C, a strain rate of 0.05 s^−1^ (equivalent strain rate of 0.02 s^−1^) and until stretching ratios of 4.76 × 4.22. The 2D strain fields for different positions along the curve have been added.

In the two previous works focused on PEF biaxial stretching [32,40], the applied settings did not allow a well-marked strain hardening development. Figure 3 represents the true stress/strain curves of the test performed in this work. The associated deformation fields for several positions along the curve have been added.

The stretching protocol based on the analysis of the master curve allows to stretch PEF up to high local strains. As can be obviously observed in the pictures, the central zone is the one that is the most deformed (Figure 3). This confirms that the heating protocol applied was efficient, allowing a weakness of the hotter central process zone. The areas close to the clamps remained colder in comparison with the central zone (Figure 3). The strain hardening confirms that the chains have been stretched (final stress values superior to 55 MPa). The onset and the slope of the strain hardening appear to be more progressive compared to what has been reported for PEF uniaxial loading performed with the same stretching protocol [35,36,37,38,39]. Compared to uniaxial conditions, simultaneous biaxial stretching in both directions is clearly less efficient regarding chain orientation and stretching in one specific direction [13].

### 3.2. Crystalline Phases Analysis

Figure S3 depicts the Debye–Scherrer pattern of an unstretched PEF sample. Before the stretching, the sample was completely amorphous: no spots or rings are observable. Accordingly, this means that all the microstructural changes observed thereafter will result only from the stretching.

**Figure 4:**
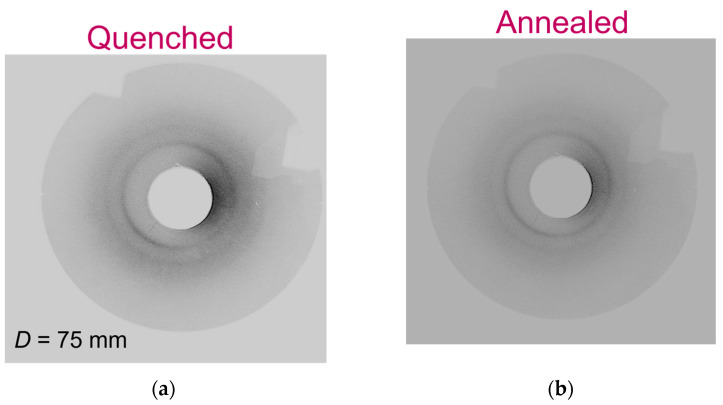
Debye-Scherrer patterns performed on PEF biaxially stretched in simultaneous and balanced conditions, followed by a quenching (**a**) or annealing (**b**) step.

Figure 4 shows the pictures of the quenched (Figure 4a) and annealed (Figure 4b) tests analyzed thanks to the Debye–Scherrer technique.

After biaxial stretching, rings are observable on the Debye–Scherrer patterns. However, this is different from the intense spots that appear after uniaxial stretching: the orientation of the crystals is less obvious in biaxial conditions. The patterns appear more similar to what is usually observed after static crystallization [39]. Even if biaxial stretching leads to a structure that can be rather assimilated to quiescent conditions, there are some differences due to the way of crystallization, such as shorter test duration, lower temperature of the test, stretching of the chains that make a quantitative comparison difficult. Nevertheless, fewer diffraction rings are present. The main hypothesis that can be proposed is the lower degree of crystallinity compared to static conditions. As less crystalline planes are present, the missing families of planes were not numerous enough to allow their observation. Crystals have been developed upon biaxial stretching, no matter the post-stretching treatment applied. The annealing step does not seem to influence the microstructure of the sample, which appears already well-defined with intense rings after the stretching end. It reveals the efficiency of the stretching settings chosen.

To obtain more descriptive data relative to the existence of crystalline phases, the Debye–Scherrer measurements were completed by DSC analyses. The DSC scans are shown in Figure S4 (in Supporting Information). The values of the recrystallization temperature (*Tc*) and crystallinity ratio (*χ_c_*) are gathered in Table 2.

Relatively high crystallinity ratios (19%) were measured in the biaxially stretched specimens for both post-stretching treatments (quenching or annealing). The crystallinity ratios reported in this work are superior to the values reported in literature [32,40] for PEF and appear comparable to what may exist for PET [50].

By performing DSC measurements at a heating rate of 1 °C/min, the maximum of the cold crystallization temperature of an amorphous sample is usually reported around 160 °C and starts around 120 °C [25]. In the case of the PEF non-stretched sample, a heating rate of 10 °C/min does not permit enough time for the chains to organize themselves in a periodic structure in the amorphous specimen: no clear crystallization or melting are visible in Figure S4. On the contrary, the cold crystallization of the stretched samples starts at lower temperature (around 100 °C), as shown in Figure S4, in relation to what is usually reported [62]. This anticipated crystallization is due to the presence of oriented areas created by the stretching that can be turned to crystalline phases. This confirms that the elongation process has an influence on the microstructural development: biaxal loading paths have allowed the chains to be extended, and oriented in the sample thickness. The furan cycles are oriented and aligned face to face with the creation of weak interactions between them, stabilized by hydrogen bonding. This bonding is going to rule and improve the order created by the stretching in the thickness of the sample.

It can be observed that for the quenched sample, the cold crystallization takes place at a lower temperature than for the annealed specimen. This feature could be due to slight relaxation occurring in some pre-ordered areas where the thermal energy provided by the heating allows for relaxation phenomena of chains that have been locally oriented upon stretching. Nevertheless, another explanation could be that the occurrence of crystallization at lower temperature is due to the presence of crystals formed upon stretching that nucleate new crystals, favoring the crystallization process. Similar conclusions were reported by van Berkel et al. [40]. Additionally, upon stretching, the melting occurs through only one single endothermal peak. This observation is different from quiescent conditions where PEF exhibits multiple melting peaks due to the presence of different crystal stabilities, or due to reorganization phenomena [24,25,29,38,62,63,64].

**Figure 5:**
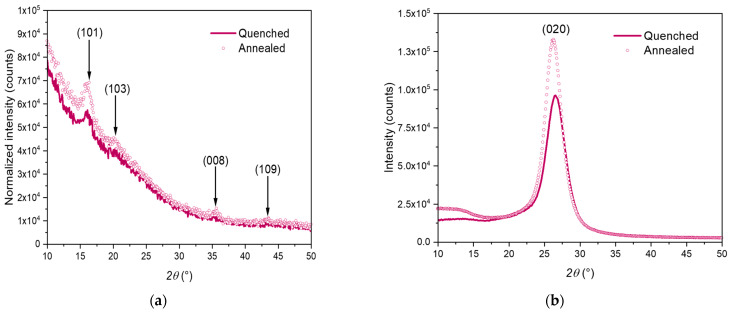
WAXS radial scans in (**a**) transmission and (**b**) reflexion mode for PEF biaxially stretched in simultaneous and balanced conditions, followed by a quenching (lines) or annealing (dots) step.

In order to have a deeper understanding of the crystal definition and of the post-stretching treatment impact, WAXS measurements were performed. Radial scans in transmission (Figure 5a) and reflexion (Figure 5b) are observable. These two scans are profiles issuing from the reading of the Debye–Scherrer patterns presented in Figure 4. Only one radial profile has been performed in one direction, and on the same sample used for the Debye–Scherrer analysis.

These scans agree with the results and conclusions already drawn with the Debye–Scherrer and DSC analyses. Whatever the post-stretching treatment (quenching or annealing), some crystalline families of planes have scattered, as shown in Figure 5.

These families of planes have been indexed by following the existing indexation of stretched PEF [33,39] (Figure 5). The structural definition of PEF monoclinic unit cell published by Mao [33,34] is the basis of the indexation protocol, arguing that PEF forms the same type of crystals both upon uniaxial and quiescent crystallization, as well as upon biaxial stretching.

Moreover, the indexation proposed in this paper fits with the angular position found in the work of Stoclet et al. for biaxially stretched sample [32]. The authors have also found the existence of the same families of planes for crystals induced upon biaxial stretching or after static crystallization.

In addition, the way of analyzing the samples (transmission or reflexion) does not allow the same families of planes to be detectable. This has been reported too in another work focused on free-blown bottles made with PET [60]. Thanks to the reflexion mode, (hk0) families of crystalline planes are observable, as these planes are parallel to the plane of the stretched film. Indeed, for PEF, the family (020) is only observable on the reflexion scan (Figure 5b), as its orientation is in the plane of the film. When the measurement is performed in transmission mode, the crystalline planes that are observed are those that are perpendicular to the specimen surface. On the other side, for a measurement performed in the reflexion mode, the planes that are parallel to the surface of the sample become visible. Mao et al. reported that the (020) family of planes could be assigned to the furan cycles that are facing each other in the stretching plane of the specimen [33,34]. As these cycles are becoming aligned in this plane, it can allow them to intensely scatter with a measurement in reflexion. The observation of different families of planes in reflexion and transmission is directly linked to the biaxial conditions that promotes stretching of the chains in two perpendicular directions at the same time. It is conceivable that the different chemicals groups are not oriented in the same direction in the thickness.

Finally, the sample that has been annealed has likely developed better defined crystalline periodicities for each of all the families referred on the graphs. Indeed, the intensity of the peaks increases, and the peaks become narrower. In comparison with uniaxial stretching conditions [33,34,39], less crystalline families are diffracted in these scans, which could be due to a lower number of crystalline planes giving diffraction. A measurement performed with a higher energy beam, e.g., Synchrotron, should reveal the existence of all the families usually reported [33,34,39].

### 3.3. Conformational Changes Observed

It has been found by Araujo et al. that the transition from amorphous PEF to crystalline PEF is associated to two conformational changes: *gauche* to *trans* for the ethylene glycol groups, and *anti* to *syn* for the furan moieties [30]. Additionally, a recent study on *SIC* proved the same conformational changes (i.e., *gauche* to *trans* and *anti* to *syn*) during uniaxial stretching [36]. The authors also highlighted the fact that the aliphatic part of the chain was strongly constrained after a uniaxial stretching, in comparison with crystallization in quiescent conditions [36].

**Figure 6:**
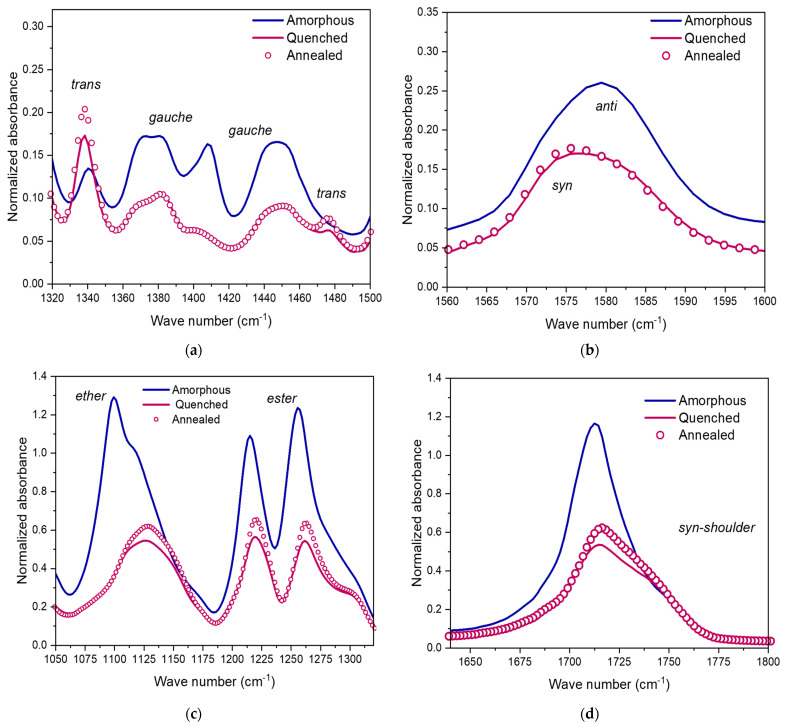
FT-IR spectra of amorphous PEF (blue line), biaxially stretched and quenched PEF (purple lines), biaxially stretched and annealed PEF (purple dots) for wave numbers associated to different chemical groups: (**a**) ethylene glycols (1320 cm^−1^ to 1500 cm^−1^), (**b**) furan cycles (1560 cm^−1^ to 1600 cm^−1^), (**c**) ether and ester groups (1050 cm^−1^ to 1320 cm^−1^), and (**d**) carbonyl area (1630 cm^−1^ to 1800 cm^−1^).

Figure 6 depicts the FTIR spectra of the annealed and quenched samples and the impact of the biaxial stretching on the conformational changes. Figure 6a focuses on the ethylene glycols vibrational zone, Figure 6b depicts rather the region of the furan cycles, while Figure 6c,d show respectively the aliphatic part (ethers and esters) and the carbonyl group stretching. Table S1 gathers the position of the vibrational bands of the associated chemical groups explored.

In the amorphous domain, the main existing conformation for the ethylene glycols is the *gauche* one, while for the furans it is the *anti* one. In comparison with the amorphous sample, the vibrational band relative to ethylene glycols in *trans* conformation increases in Figure 6a, for both quenched and annealed stretched samples, whereas the signal of the *gauche* conformation decreases with the stretching. As shown in Figure 6b, in comparison with amorphous PEF, there is a shift of the maximum of the band that represents the furan in *anti* conformation (1580 cm^−1^) to the band relative to the furans in *syn* conformation (1575 cm^−1^). It was also noticeable for cold crystallized PEF samples [30,36].

Moreover, when the amorphous and biaxially stretched samples are compared in the region of the ether and ester groups (Figure 6c), it appears that the stretching has led to a high constrain of these groups: their vibration is occurring at highest wave numbers for the biaxially stretched specimens. It is associated to a highest energy. Figure 6d points out some differences in the position of the maximum of the carbonyl groups band for the stretched samples in comparison to the amorphous one: there is a shift to the right part of the spectra, which is associated to a higher vibrational energy of this group. This shift testifies that the carbonyl groups were mainly in *anti* conformation before the stretching and are in *syn* conformation after the biaxial loading. Moreover, the apparition of a pronounced shoulder around 1740 cm^−1^ for all the stretched samples confirms the highest constraint of this group in comparison with an amorphous sample. In a nutshell, *SIC* occurrence upon biaxial stretching leads to similar changes of conformation as the ones reported over uniaxial stretching. This feature can show that the crystals induced by a mechanical stretching are equivalent for biaxial and uniaxial tests, but also the same as the one induced during quiescent crystallization [30,36].

Stoclet et al. have proposed that the orientation degree of the chains is the parameter that is controlling the strain induced crystallization upon biaxial stretching. On the other side, the extension of the amorphous chain monitors the mechanical behavior [32]. They claim that the slope of the strain hardening is governed by the orientation of the amorphous domain. The chain extension would be the parameter controlling the strain hardening. Our results do agree with these conclusions, where biaxial stretching promotes the extension of the chains (change from *gauche* to *trans* conformations) which may have induced strain-hardening of the material upon stretching. A high level of constraint consequently does exist in the amorphous phase.

If the annealed and quenched samples are compared, the wave numbers remain the same for all the explored bands but for some groups the height is changing. The annealed sample exhibits, most of the time, the highest height (Figure 6a,b,d). This could be a proof of a more stabilized microstructure due to the annealing step. This feature appears only for the groups localized in the aliphatic part, while the furan seems to not be impacted by the annealing, having already been stabilized by the stretching.

### 3.4. Amorphous Phase Mobility

**Figure 7:**
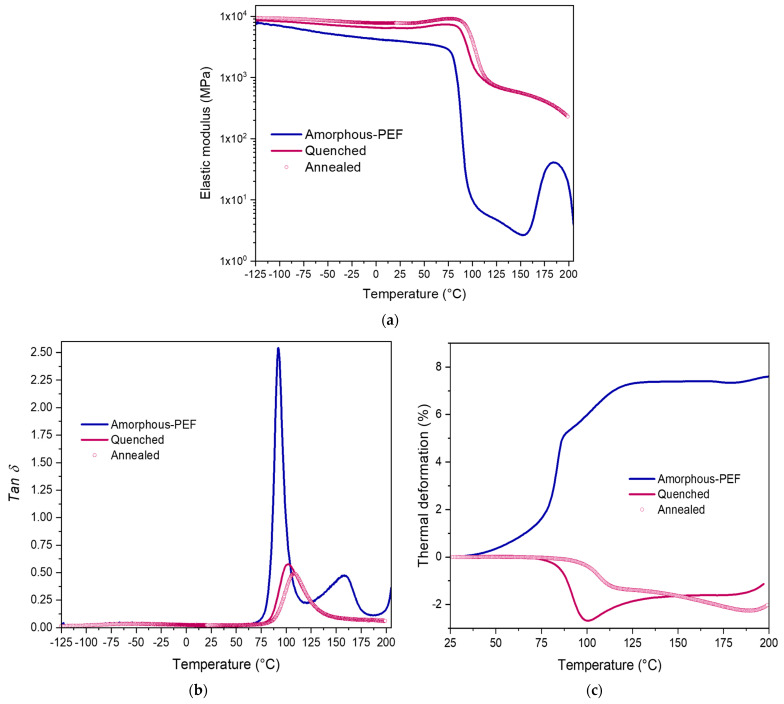
DMTA run of amorphous and biaxially stretched PEF carried out at 1 °C/min and 1 Hz, depicting the evolution of (**a**) the elastic modulus (*E′*), (**b**) *Tan δ* for temperatures ranging from −125 °C to 210 °C and (**c**) the thermal deformation for temperatures varying from 25 °C to 200 °C.

The post-stretching stiffness, α-relaxation temperature, and thermal stability of the stretched samples were analyzed by DMTA measurements, as displayed in Figure 7.

The DMTA scan of the amorphous sample (blue line) reveals an *α*-relaxation around 80 °C, and the occurrence of the cold crystallization around 150 °C. The cold crystallization is observable in DMTA (Figure 7) and not in DSC (Figure S4) because the heating rate is slower in DMTA (1 °C/min). With DSC, the heating rate is faster (10 °C/min) so that the chains do not have enough molecular mobility to develop crystallization processes.

The comparison of the DMTA scans of the amorphous and the biaxially stretched samples confirm what has been found with DSC analysis. Indeed, the amorphous phase of the stretched samples has a different mobility than the amorphous sample, as it is visible reflected in the higher *α*-relaxation temperature and the better thermal stability. The absence of cold crystallization for the biaxially stretched samples in comparison with the amorphous sample is due to the stretching that has already induced a strong crystal upon stretching (there is less amorphous domain in the stretched sample), and a high rigidity of the amorphous domain. Additionally, the cold crystallization of the stretched samples is visible in DSC (Figure S4), but not with the DMTA measurement (Figure 7). DSC and DMTA are two measurements that are complementary in polymer sciences, but they do not measure the transition on the same basis. DSC measurement relies on thermal exchange, while DMTA reports the evolution of the rigidity for a certain frequency and during a temperature scan. As the stretched samples are already crystalline and stiff, the occurrence of cold crystallization could not induce a noticeable change in the sample rigidity. On the other side, with DSC the exothermic phenomenon is detected.

A similar thermo-mechanical behavior exists for the two biaxially stretched samples, whatever the post-stretching treatment (quenching or annealing). The stretching has clearly promoted an increase of the elastic modulus, in both the glassy and rubbery plateaux. The obvious increase of *T_α_* testifies the presence of the crystalline phases induced upon biaxial tests. The low magnitude of the *Tan δ* peak of the two biaxially stretched specimens confirms that the amorphous domain of these two samples does not have a high mobility because of the presence of the crystallinity.

Additionally, the annealed sample appears more stable, as its *α*-relaxation temperature is reported at 108 °C, while the other one occurs at 103 °C. The annealing step has led to an amorphous domain that present chains with restricted motions. These *α*-transition temperatures are similar to what has been reported for PEF loaded under biaxial conditions [40], and to what has been reported after an efficient uniaxial stretching [35].

Moreover, it can be noticed that the annealed sample is more rigid than the quenched one, as the glassy plateau is localized above that of the quenched sample. After the annealing step, the thermal stability is improved in comparison with the sample quenched (Figure 7c). In fact, the imposed annealing step has authorized the chains to release mechanical stresses. By the way, during DMTA measurement, the chain relaxation is visible at higher temperatures for the annealed samples, in relation to the chains of the quenched specimens that have been frozen in the state induced by the stretching.

All these results confirm that PEF can develop a microstructure with a high stability and a well-organized microstructure after biaxial stretching. The stretchability of the material upon large deformations, and especially in blowing processes such ISBM, appears promising.

## 4. Conclusions

This work has shown that the protocol defined, based on time/temperature equivalence principle, was efficient to identify stretching conditions suitable for biaxial tests. The tests performed were simultaneous and balanced. Moreover, thanks to the master curve building, the stretching was performed on samples that were in a rubbery-like state. By the way, the reaching of high draw ratios was possible. A strain hardening was observed. The samples exhibit high crystallinity ratios and a good stability of their microstructure. This work proves that crystals have been created upon PEF biaxial stretching thanks to strain induced crystallization in a control way. The stretching and the presence of crystals have an influence on the mobility of the amorphous domain that is more rigid. The influence of an annealing step after the stretching was observed. The annealing step induces the formation of a higher amount of organized phase whose thermal stability is enhanced. Nevertheless, once the crystals are built, the same families of crystalline planes exist as those reported for uniaxial stretching and quiescent crystallization. This reveals that the polymer crystallizes in the same crystalline structure whatever the type of stretching performed (uniaxial and biaxial). The crystalline structure developed upon mechanical solicitation and strain induced crystallization also seems to be similar to that induced upon quiescent conditions. However, the conformational changes occurring upon biaxial stretching are similar to those that exist upon uniaxial stretching, and the aliphatic part of the chain remains highly constrained by the mechanical loading.

## Figures and Tables

**Table 1:** Biaxial draw ratio and values of the evolution of the true longitudinal (*ε_xx_*), transversal(*ε_yy_*), major (*ε*_1_), and minor (*ε*_2_) strains.

*ε_xx_*	*ε_yy_*	*ε*_1_	*ε*_2_	*λ_diagonal_*_1_	*λ_diagonal_*_2_	*λ_biaxial_*
1.52	1.48	1.56	1.44	4.71	4.66	21.97

**Table 2:** *χ_c_* and *T_c_* of biaxially stretched specimens.

Samples	*χ_c_* (%)	*T_c_* (°C)
Amorphous PEF	0	
A-quenched	19	111
A-annealed	19	120

## Supplementary Materials

The following supporting information can be downloaded at: https://www.mdpi.com/article/10.3390/polym15030661/s1. Figure S1. Geometry of the biaxial samples. Dimensions are in mm. R5 is the curvature radius; Figure S2. Evolution of the true longitudinal strain (Hencky’s strain) with the time on the two diagonals, (b) evolution of the true longitudinal (*ε_xx_*), transversal (*ε_yy_*), major (*ε*_1_), and minor (*ε*_2_) strains; Figure S3. Debye-Scherrer pattern of unstretched PEF; Figure S4. Thermal behaviour of annealed (dots) and quenched (lines) samples, measured by DSC scans performed at 10 °C/min from 70 °C to 250 °C. Endothermic phenomena are top-down; Table S1. Values of the vibrational bands explored with the associated chemical groups.

## References

[1] Gandini A. (2011). The Irruption of Polymers from Renewable Resources on the Scene of Macromolecular Science and Technology. Green Chem..

[2] Gandini A., Lacerda T.M., Carvalho A.J.F., Trovatti E. (2016). Progress of Polymers from Renewable Resources: Furans, Vegetable Oils, and Polysaccharides. Chem. Rev..

[3] Gandini A., Silvestre A.J.D., Neto C.P., Sousa A.F., Gomes M. (2009). The Furan Counterpart of Polyethylene Terephthalate: An Alternative Material Based on Renewable Resources. J. Polym. Sci. Polym. Chem..

[4] Papageorgiou G.Z., Papageorgiou D.G., Terzopoulou Z., Bikiaris D.N. (2016). Production of Bio-Based 2,5-Furan Dicarboxylate Polyesters: Recent Progress and Critical Aspects in Their Synthesis and Thermal Properties. Eur. Polym. J..

[5] Sousa A.F., Coelho J.F.J., Silvestre A.J.D. (2016). Renewable-Based Poly((Ether)Ester)s from 2,5-Furandicarboxylic Acid. Polymer.

[6] Matos M., Sousa A.F., Fonseca A.C., Freire C.S.R., Coelho J.F.J., Silvestre A.J.D. (2014). A New Generation of Furanic Copolyesters with Enhanced Degradability: Poly(Ethylene 2,5-Furandicarboxylate)-Co-Poly(Lactic Acid) Copolyesters. Macromol. Chem. Phys..

[7] Loos K., Zhang R., Pereira I., Agostinho B., Hu H., Maniar D., Sbirrazzuoli N., Silvestre A.J.D., Guigo N., Sousa A.F. (2020). A Perspective on PEF Synthesis, Properties, and End-Life. Front. Chem..

[8] Sousa A.F., Vilela C., Fonseca A.C., Matos M., Freire C.S.R., Gruter G.-J.M., Coelho J.F.J., Silvestre A.J.D. (2015). Biobased Polyesters and Other Polymers from 2,5-Furandicarboxylic Acid: A Tribute to Furan Excellency. Polym. Chem..

[9] Guigo N., Forestier E., Sbirrazzuoli N. (2019). Thermal Properties of Biobased Polymers: Furandicarboxylic Acid (FDCA)-Based Polyesters.

[10] Sousa A.F., Patrício R., Terzopoulou Z., Bikiaris D.N., Stern T., Wenger J., Loos K., Lotti N., Siracusa V., Szymczyk A. (2021). Recommendations for Replacing PET on Packaging, Fiber, and Film Materials with Biobased Counterparts. Green Chem..

[11] De Jong E., Visser H.R.A., Dias A.S., Harvey C., Gruter G.-J.M. (2022). The Road to Bring FDCA and PEF to the Market. Polymers.

[12] Gandini A., Lacerda T.M. (2022). Furan Polymers: State of the Art and Perspectives. Macromol. Mater. Eng..

[13] Billon N., Picard M., Gorlier E. (2014). Stretch Blow Moulding of PET; Structure Development and Constitutive Model. Int. J. Mater. Form..

[14] Menary G.H., Tan C.W., Harkin-Jones E.M.A., Armstrong C.G., Martin P.J. (2012). Biaxial Deformation and Experimental Study of PET at Conditions Applicable to Stretch Blow Molding. Polym. Eng. Sci..

[15] Burgess S.K., Leisen J.E., Kraftschik B.E., Mubarak C.R., Kriegel R.M., Koros W.J. (2014). Chain Mobility, Thermal, and Mechanical Properties of Poly(Ethylene Furanoate) Compared to Poly(Ethylene Terephthalate). Macromolecules.

[16] Dimitriadis T., Bikiaris D.N., Papageorgiou G.Z., Floudas G. (2016). Molecular Dynamics of Poly(Ethylene-2,5-Furanoate) (PEF) as a Function of the Degree of Crystallinity by Dielectric Spectroscopy and Calorimetry. Macromol. Chem. Phys..

[17] Koros W.J., Burgess S.K., Chen Z. (2015). Polymer Transport Properties. Encyclopedia of Polymer Science and Technology.

[18] Burgess S.K., Kriegel R.M., Koros W.J. (2015). Carbon Dioxide Sorption and Transport in Amorphous Poly(Ethylene Furanoate). Macromolecules.

[19] Burgess S.K., Wenz G.B., Kriegel R.M., Koros W.J. (2016). Penetrant Transport in Semicrystalline Poly(Ethylene Furanoate). Polymer.

[20] Burgess S.K., Karvan O., Johnson J.R., Kriegel R.M., Koros W.J. (2014). Oxygen Sorption and Transport in Amorphous Poly(Ethylene Furanoate). Polymer.

[21] Burgess S.K., Mikkilineni D.S., Yu D.B., Kim D.J., Mubarak C.R., Kriegel R.M., Koros W.J. (2014). Water Sorption in Poly(Ethylene Furanoate) Compared to Poly(Ethylene Terephthalate). Part 1: Equilibrium Sorption. Polymer.

[22] Burgess S.K., Mikkilineni D.S., Yu D.B., Kim D.J., Mubarak C.R., Kriegel R.M., Koros W.J. (2014). Water Sorption in Poly(Ethylene Furanoate) Compared to Poly(Ethylene Terephthalate). Part 2: Kinetic Sorption. Polymer.

[23] Bourdet A., Esposito A., Thiyagarajan S., Delbreilh L., Affouard F., Knoop R.J.I., Dargent E. (2018). Molecular Mobility in Amorphous Biobased Poly(Ethylene 2,5-Furandicarboxylate) and Poly(Ethylene 2,4-Furandicarboxylate). Macromolecules.

[24] Stoclet G., Arias A., Yeniad B., de Vos S. (2019). Relationships between Crystalline Structure and the Thermal Behavior of Poly(Ethylene 2,5-furandicarboxylate): An in Situ Simultaneous SAXS-WAXS Study. Polym. Eng. Sci..

[25] Stoclet G., Gobius du Sart G., Yeniad B., de Vos S., Lefebvre J.M. (2015). Isothermal Crystallization and Structural Characterization of Poly(Ethylene-2,5-Furanoate). Polymer.

[26] Knoop R.J.I., Vogelzang W., van Haveren J., van Es D.S. (2013). High Molecular Weight Poly(Ethylene-2,5-Furanoate); Critical Aspects in Synthesis and Mechanical Property Determination. J. Polym. Sci. Polym. Chem..

[27] Papageorgiou G.Z., Tsanaktsis V., Bikiaris D.N. (2014). Synthesis of Poly(Ethylene Furandicarboxylate) Polyester Using Monomers Derived from Renewable Resources: Thermal Behavior Comparison with PET and PEN. Phys. Chem. Chem. Phys..

[28] Maini L., Gigli M., Gazzano M., Lotti N., Bikiaris D., Papageorgiou G. (2018). Structural Investigation of Poly(Ethylene Furanoate) Polymorphs. Polymers.

[29] Tsanaktsis V., Papageorgiou D.G., Exarhopoulos S., Bikiaris D.N., Papageorgiou G.Z. (2015). Crystallization and Polymorphism of Poly(Ethylene Furanoate). Cryst. Growth Des..

[30] Araujo C.F., Nolasco M.M., Ribeiro-Claro P.J.A., Rudić S., Silvestre A.J.D., Vaz P.D., Sousa A.F. (2018). Inside PEF: Chain Conformation and Dynamics in Crystalline and Amorphous Domains. Macromolecules.

[31] Stoclet G., Lefebvre J.M., Yeniad B., Gobius du Sart G., de Vos S. (2018). On the Strain-Induced Structural Evolution of Poly(Ethylene-2,5-Furanoate) upon Uniaxial Stretching: An in-Situ SAXS-WAXS Study. Polymer.

[32] Stoclet G., Xu S., Gaucher V., Tahon J.F., van Berkel S., Arias A., Rogeret C., Nourichard R., de Vos S. (2021). Influence of the Molecular Weight on Mechanical Behavior and Associated Strain-Induced Structural Evolution of Poly(Ethylene 2,5-Furandicarboxylate) upon Biaxial Stretching. Polymer.

[33] Mao Y., Kriegel R.M., Bucknall D.G. (2016). The Crystal Structure of Poly(Ethylene Furanoate). Polymer.

[34] Mao Y., Bucknall D.G., Kriegel R.M. (2018). Synchrotron X-Ray Scattering Study on Amorphous Poly(Ethylene Furanoate) under Uniaxial Deformation. Polymer.

[35] Forestier E., Combeaud C., Guigo N., Sbirrazzuoli N. (2022). A Proposal for Enhanced Microstructural Development of Poly(Ethylene 2,5-Furandicarboxylate), PEF, upon Stretching: On Strain-Induced Crystallization and Amorphous Phase Stability Improvement. Polymer.

[36] Forestier E., Guigo N., Combeaud C., Billon N., Sbirrazzuoli N. (2020). Conformational Change Analysis of Poly(Ethylene 2,5-Furandicarboxylate) and Poly(Ethylene Terephthalate) under Uniaxial Stretching. Macromolecules.

[37] Forestier E., Combeaud C., Guigo N., Sbirrazzuoli N., Billon N. (2020). Understanding of Strain-Induced Crystallization Developments Scenarios for Polyesters: Comparison of Poly(Ethylene Furanoate), PEF, and Poly(Ethylene Terephthalate), PET. Polymer.

[38] Forestier E., Combeaud C., Guigo N., Corvec G., Pradille C., Sbirrazzuoli N., Billon N. (2021). Comparative Analysis of the Mechanical Behaviour of PEF and PET Uniaxial Stretching Based on the Time/Temperature Superposition Principle. Polymers.

[39] Forestier E., Combeaud C., Guigo N., Monge G., Haudin J.M., Sbirrazzuoli N., Billon N. (2020). Strain-Induced Crystallization of Poly(Ethylene 2,5-Furandicarboxylate). Mechanical and Crystallographic Analysis. Polymer.

[40] van Berkel J.G., Guigo N., Kolstad J.J., Sbirrazzuoli N. (2018). Biaxial Orientation of Poly(Ethylene 2,5-Furandicarboxylate): An Explorative Study. Macromol. Mater. Eng..

[41] Lofgren E.A., Jabarin S.A. (1994). Polarized Internal Reflectance Spectroscopic Studies of Oriented Poly(Ethylene Terephthalate). J. Appl. Polym. Sci..

[42] Chandran P., Jabarin S. (1993). Biaxial Orientation of Poly(Ethylene Terephthalate). Part III: Comparative Structure and Property Changes Resulting from Simultaneous and Sequential Orientation. Adv. Polym. Technol..

[43] Quandalle G. (2017). Study and Mechanical Modeling of the Strain-Induced-Crystallization of Polymers: Crosslinked Naturel Rubber and PET. Ph.D. Thesis.

[44] Menary G.H., Tan C.W., Armstrong C.G. (2012). The Effect of Temperature, Strain Rate and Strain on the Induced Mechanical Properties of Biaxially Stretched PET. Key Eng. Mater..

[45] Gohil R.M., Schultz J.M. (1993). Morphology of Biaxially Stretched Poly(Ethylene Terephthalate) Films. J. Macromol. Sci. Part B.

[46] Maruhashi Y., Asada T. (1996). Structure and Properties of Biaxially Stretched Poly(Ethylene Terephthalate) Sheets. Polym. Eng. Sci..

[47] Bandla S., Allahkarami M., Hanan J.C. (2014). Out-of-Plane Orientation and Crystallinity of Biaxially Stretched Polyethylene Terephthalate. Proceedings of the Powder Diffraction.

[48] Chandran P., Jabarin S. (1993). Biaxial Orientation of Poly(Ethylene Terephthalate). Part I: Nature of the Stress—Strain Curves. Adv. Polym. Technol..

[49] Marco Y., Chevalier L., Régnier G., Poitou A. (2002). Induced Crystallization and Orientation of Poly(Ethylene Terephthalate) during Uniaxial and Biaxial Elongation. Proceedings of the Macromolecular Symposia.

[50] Marco Y., Chevalier L., Chaouche M. (2002). WAXD Study of Induced Crystallization and Orientation in Poly(Ethylene Terephthalate) during Biaxial Elongation. Polymer.

[51] Gohil R.M., Salem D.R. (1993). Orientation Distribution in the Noncrystalline Regions of Biaxially Drawn Poly(Ethylene Terephthalate) Film: A Chain-intrinsic Fluorescence Study. J. Appl. Polym. Sci..

[52] Spruiell J.E., White J.L. (1986). Structural Characterization of Crystallinity and Crystalline Orientation in Simultaneously Biaxially Stretched and Annealed Polyethylene Terephthalate Films. J. Polym. Eng..

[53] Bower D.I., Jarvis D.A., Ward I.M. (1986). Molecular Orientation in Biaxially Oriented Sheets of Poly(Ethylene Terephthalate). I. Characterization of Orientation and Comparison with Models. J. Polym. Sci. B Polym. Phys..

[54] Zekriardehani S., Jabarin S.A., Gidley D.R., Coleman M.R. (2017). Effect of Chain Dynamics, Crystallinity, and Free Volume on the Barrier Properties of Poly(Ethylene Terephthalate) Biaxially Oriented Films. Macromolecules.

[55] Hassan M., Cakmak M. (2013). Mechano Optical Behavior of Polyethylene Terephthalate Films during Simultaneous Biaxial Stretching: Real Time Measurements with an Instrumented System. Polymer.

[56] Everall N., MacKerron D., Winter D. (2002). Characterisation of Biaxial Orientation Gradients in Poly(Ethylene Terephthalate) Films and Bottles Using Polarised Attenuated Total Reflection FTIR Spectroscopy. Polymer.

[57] Cakmak M., Spruiell J.E., White J.L., Lin J.S. (1987). Small Angle and Wide Angle X-Ray Pole Figure Studies on Simultaneous Biaxially Stretched Poly(Ethylene Terephthalate) (PET) Films. Polym. Eng. Sci..

[58] Hassan M.K., Cakmak M. (2015). Strain-Induced Crystallization during Relaxation Following Biaxial Stretching of PET Films: A Real-Time Mechano-Optical Study. Macromolecules.

[59] Jarvis D.A., Hutchinson I.J., Bower D.I., Ward I.M. (1980). Characterization of Biaxial Orientation in Poly(Ethylene Terephthalate) by Means of Refractive Index Measurements and Raman and Infra-Red Spectroscopies. Polymer.

[60] Picard M. (2008). Strain Induced Crystallisation during Stretch Blow Moulding of PET; Correlation with Strain Hardening. Ph.D. Thesis.

[61] Al-Itry R., Lamnawar K., Maazouz A., Billon N., Combeaud C. (2015). Effect of the Simultaneous Biaxial Stretching on the Structural and Mechanical Properties of PLA, PBAT and Their Blends at Rubbery State. Eur. Polym. J..

[62] Righetti M.C., Vannini M., Celli A., Cangialosi D., Marega C. (2022). Bio-Based Semi-Crystalline PEF: Temperature Dependence of the Constrained Amorphous Interphase and Amorphous Chain Mobility in Relation to Crystallization. Polymer.

[63] Van Berkel J.G., Guigo N., Kolstad J.J., Sipos L., Wang B., Dam M.A., Sbirrazzuoli N. (2015). Isothermal Crystallization Kinetics of Poly (Ethylene 2,5-Furandicarboxylate). Macromol. Mater. Eng..

[64] Papageorgiou G.Z., Nikolaidis G.N., Ioannidis R.O., Rinis K., Papageorgiou D.G., Klonos P.A., Achilias D.S., Kapnisti M., Terzopoulou Z., Bikiaris D.N. (2022). A Step Forward in Thermoplastic Polyesters: Understanding the Crystallization and Melting of Biobased Poly(Ethylene 2,5-Furandicarboxylate) (PEF). ACS Sustain. Chem. Eng..

