# Supplementary material for: Mechanical Behaviour and Induced Microstructural Development upon Simultaneous and Balanced Biaxial Stretching of Poly(ethylene furandicarboxylate), PEF
Source: Polymers (Basel). 2023 Jan 28;15(3):661. doi: 10.3390/polym15030661 (PMC9919262; doi:10.3390/polym15030661)
Supplement: Supplementary file 1 [file polymers-15-00661-s001.zip › polymers-2097800-supplementary.pdf]

Supporting Information

1. Mechanical behaviour

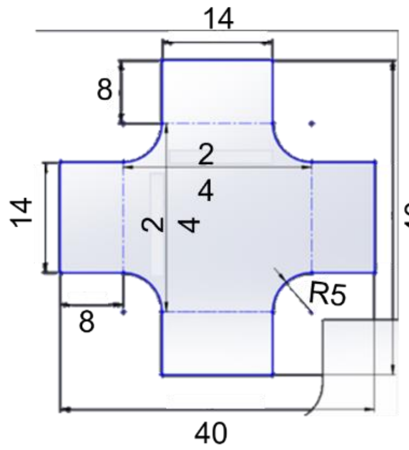

Figure S1. Geometry of the biaxial samples. Dimensions are in mm. R5 is the curvature radius.

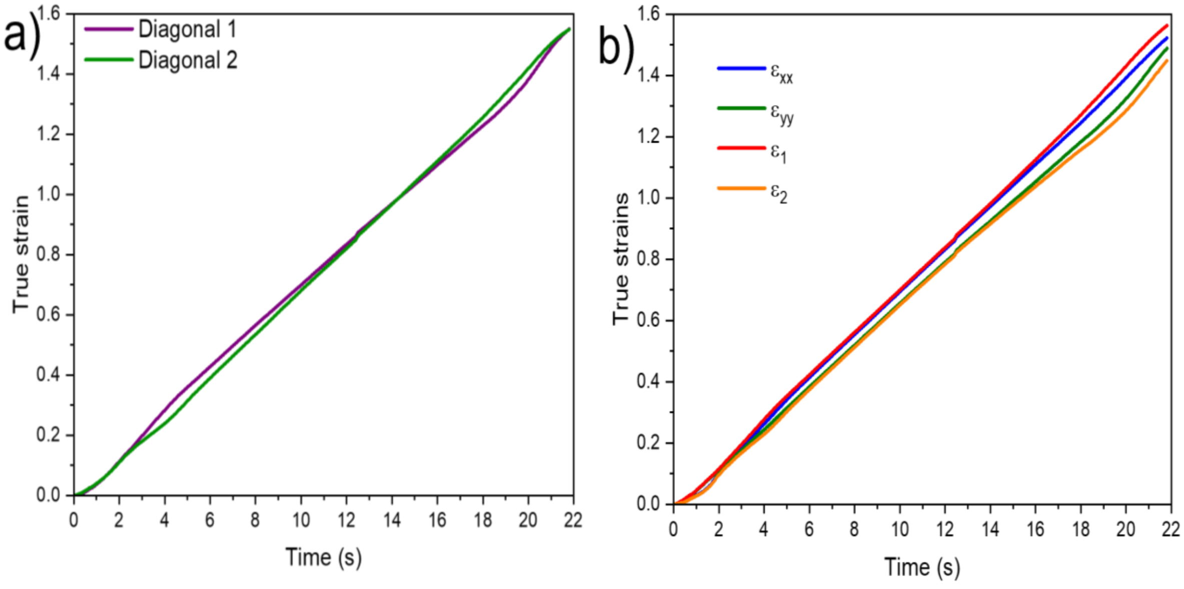

Figure S2. Evolution of the true longitudinal strain (Hencky's strain) with the time on the two diagonals, (b) evolution of the true longitudinal (ε_{xx}), transversal (ε_{yy}), major (ε_1), and minor (ε_2) strains.

The correctness of the test is visible with the equivalence of the measurement on both diagonals (Figure S2b). The quality of the control of the strain rate is visible by the linearity of the evolution for PEF. The closeness of the strain curves in Figure S2b confirms the quality of the biaxial loading in the central zone.

2. *Microstructural analysis*

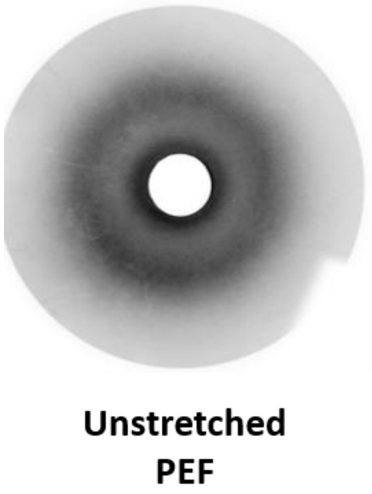

Figure S3. Debye-Scherrer pattern of unstretched PEF.

3. *Thermal analysis*

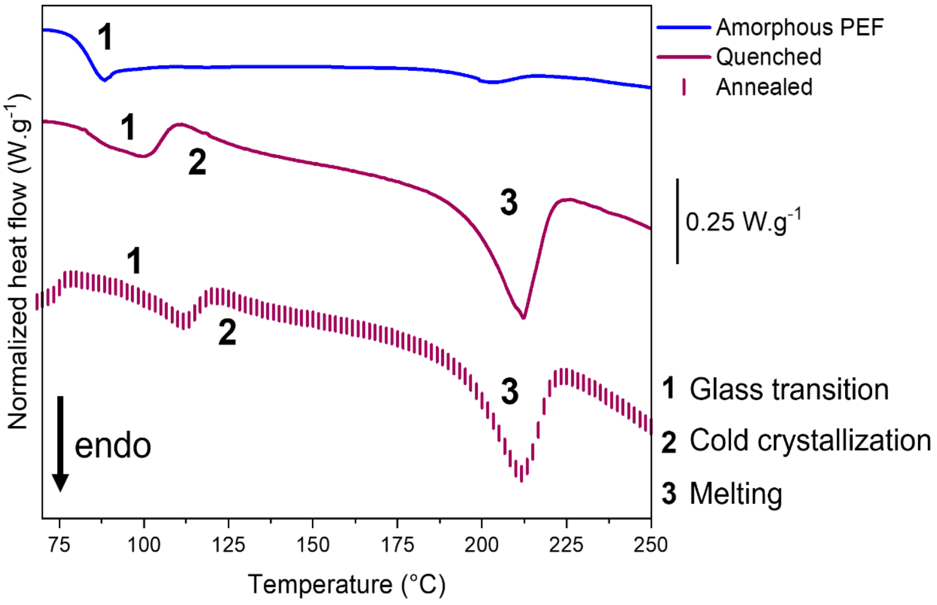

Figure S4. Thermal behaviour of annealed (dots) and quenched (lines) samples, measured by DSC scans performed at 10 °C/min from 70 °C to 250 °C. Endothermic phenomena are top-down.

4. Conformational analysis

Band (cm^{-1})	Chemical group
1115	***C-O***-*C (stretching)*
1225-1275	*O=C-**O-C** (stretching)*
1340	*trans*^{EG} *(wagging)*
1380	*gauche*^{EG} *(wagging)*
1460	*gauche*^{EG} *(bending)*
1480	*trans*^{EG} *(bending)*
1576	*syn*^{FDCA} *(stretching)*
1580	*anti*^{FDCA} *(stretching)*
1718	*C=O (stretching)*
1732	*C=O (syn-shoulder)*
3120 / 3160	*=C-H (furan)*

Table S1. Values of the vibrational bands explored with the associated chemical groups.
